# The association between community mental health nursing and hospital admissions for people with serious mental illness: a systematic review

**DOI:** 10.1186/s13643-020-01292-y

**Published:** 2020-02-17

**Authors:** Matthew J. Leach, Martin Jones, Dan Bressington, Adrian Jones, Fiona Nolan, Kuda Muyambi, Marianne Gillam, Richard Gray

**Affiliations:** 1 Department of Rural Health, University of South Australia, North Terrace, Adelaide, South Australia 5000 Australia; 2 Department of Rural Health, University of South Australia, 111 Nicholson Avenue, Whyalla Norrie, South Australia 5608 Australia; 3 School of Nursing, Hong Kong Polytechnic University, Hung Hom, Kowloon, Hong Kong, SAR China; 4 Betsi Cadwaladr University Health Board, Wrexham Maelor Hospital, Wrexham, Wales, LL167TD UK; 5 Florence Nightingale Foundation, School of Health and Human Science, University of Essex, Wivenhoe Park, Colchester, Essex, CO4 3SQ UK; 6 School of Nursing and Midwifery, La Trobe University, Bundoora, Victoria 3086 Australia

**Keywords:** Hospital admission, Community mental health nursing, Mental illness, Relapse, Systematic review

## Abstract

**Background:**

Relapse prevention is an important objective in the management of serious mental illness (SMI). While community mental health nurses (CMHN) might be well-placed to support people with SMI in averting relapse, no systematic reviews have examined this association.

**Aim:**

To review the evidence from studies reporting an association between CMHN exposure and hospitalisation of persons living with SMI (a proxy for relapse).

**Methods:**

Searches were undertaken in ten bibliographic databases and two clinical trial registries. We included studies of patients with SMI, where CMHN was the exposure, and the outcome was relapse (i.e. readmission to a psychiatric inpatient facility). Quality assessment of included studies was completed using two risk-of-bias measures.

**Results:**

Two studies met the inclusion criteria. Studies were rated as being of low-moderate methodological quality. There was insufficient evidence to conclude that community mental health nursing reduced the risk of admission to psychiatric inpatient facilities.

**Conclusions:**

The review found no evidence that CMHN was associated with higher or lower odds of admission to psychiatric inpatient facilities among patients with SMI. The findings of the review point to a need for further research to investigate the impact of CMHN exposure and relapse in people with SMI.

**Systematic review registration:**

PROSPERO CRD42017058694

## Background

Mental illness is a global health priority, with at least 10% of the world’s population affected by a psychiatric disorder at any one time [1]. The global cost of managing these disorders approximated US$2.5 trillion in 2010, with expenditure expected to reach US$6.1 trillion by 2030 [2]. The sizeable cost of mental illness, together with the high prevalence and elevated risk of physical morbidity and mortality in this population [3], contribute to considerable disease burden. In fact, mental illness attributes to 32% of global disease burden in terms of years lived with disability—more than any other condition [4].

Mental disorders vary in severity, from mild disturbances in thought and/or behaviour, to more serious mental illness (SMI), such as schizophrenia, bipolar disorder and depression with psychotic features [5]. SMI represents a group of non-organic psychotic disorders that are both persistent (i.e. has a duration of treatment of 2 years or more) and contribute to demonstrable dysfunction [6]. Compared with the general population, people diagnosed with a SMI have a 1.4–2.0 times higher risk of cardiovascular disease [5] and are more likely to be hospitalised [7], present to the emergency department (2.9-fold increased risk) [8] and be victims of crime (2.3 to 140-fold increased risk) [9]. Effective support and management of SMI are essential to reducing the burden of serious mental illness [10].

Over the past two decades, there has been a strong emphasis on the use of community-based services models (e.g. crisis teams, early intervention services, case management) to manage serious mental illness [11]. A primary aim of these models is to offer intensive support at home rather than admission to hospital, and when admission to hospital does occur, to facilitate early discharge [12]. The focus on relapse prevention is an important clinical outcome for patients with SMI. Not only does preventing relapse reduce the risk of future relapses, it may also enhance quality of life and reduce distress for the patient [13]. Furthermore, relapse is associated with considerable cost to the health system [14]. For example, in the United Kingdom (UK), it has been estimated that treatment costs associated with relapse in the previous 6 months are at least four times higher than those for patients who have not relapsed [15].

Community mental health nurses are appropriately placed to avert relapse in people living with SMI. Anecdotal reports from groups of stakeholders in the UK have indicated that community mental health nurses have more face-to-face contact with individuals living with SMI relative to other disciplines [16]. Hence, their potential to impact the clinical outcomes of people with SMI may be substantial [17, 18].

We found only one previous systematic review of the effectiveness of community mental health nurses [19]. This review was published almost 25 years ago and included 11 trials. Most included studies focused on the testing of specific interventions (e.g. family work) and not the impact of community mental health nursing as the exposure of interest. The authors concluded that their review did little to refute the idea that the efficacy of community mental health nursing in relation to patient outcomes may something of a myth. There has been no subsequent systematic review of the literature on this topic. Other clinical disciplines (such as midwifery) [20] have established a strong empirical case, clearly demonstrating—in meta-analyses—better outcomes compared with usual care. In mental health, a similar evidence base is important in informing and planning models of service delivery.

## Methods

### Aim

This systematic review aimed to investigate the association between exposure to community mental health nursing care and admission to hospital (a suitable proxy for relapse) [20] in people with serious mental illness.

### Study selection process

Observational studies (including case-control, cohort and cross-sectional studies) and clinical trials (including non-randomised controlled trials and randomised controlled trials) [i.e. study design] examining the effect of care provided by mental health nurses [i.e. exposure] to community-dwelling patients with SMI (i.e. schizophrenia, bipolar disorder and major depression) [i.e. participants] were eligible for inclusion in this review. The intervention could be compared with any other model of care [i.e. comparator]. For this review, we defined a community mental health or psychiatric nurse as a person holding a formal specialised qualification in psychiatric/mental health nursing and had been registered, credentialed or licenced to practise in that capacity (e.g. registered mental health/psychiatric nurse) and was working primarily in the community. It is important to note that while the review protocol did not explicitly mention ‘community’ mental health nurses, it was implied. Studies evaluating multidisciplinary team-based models of care, specific mental health nurse administered clinical interventions (e.g. family work, cognitive behavioural therapy) or care provided in secure or other inpatient settings were excluded. No restriction was applied to the language or date of publication.

The MEDLINE [Ovid, 1946 to present] search strategy is presented in Table 1. This strategy was adapted, as necessary, for the following databases: CINAHL [EBSCOHost, 1937 to present], PubMed [NCBI, 1966 to present], EMBASE [Ovid, 1947 to present], Nursing & Allied Health Database [ProQuest, inception to present], Health Source: Nursing/Academic Edition [EBSCOHost, inception to present], PsycINFO [Ovid, 1806 to present], Ovid Nursing [Ovid, 1946 to present], ProQuest Dissertations and Theses Global [ProQuest, 1743 to present], The Cochrane Library [1992 to present], and Web of Science [Clarivate Analytics, 1975 to present]. All databases were searched from the date of inception to June 2017. The reference lists of included publications, and articles citing the included publications, were also hand searched to identify potentially eligible studies. Clinical trial registries (i.e. clinicaltrials.gov; WHO Clinical Trials) were searched to identify any on-going or unpublished trials. The search strategy was initially implemented in July 2017 and updated in November 2019.

**Table 1:** MEDLINE search strategy

i. Mental health nurs$ [mp] OR psychiatric nurs$ [mh,mp] ii. Severe mental illness [mp] OR mental disorders [mh] OR mental illness [mp] OR schizo$ [mh,mp] OR bipolar disorder [mh] OR psychos?s [mp] OR psychotic [mp] OR psychotic affective disorders [mh] OR psychotic disorders [mh] OR psychotic depression [mp] iii. Patient admission [mh] OR patient readmission [mh] OR hospital admission [mp] OR hospital readmission [mp] OR unplanned readmission [mp] OR hospitali?ation [mp] OR readmission rate [mp] OR length of stay [mh] OR emergency department presentation [mp] OR admission to home treatment [mp] OR access to crisis intervention [mp] OR drop-in treatment [mp] OR drop-in care [mp] OR drop-in unit [mp] OR drop-in centre [mp] OR home intervention [mp] OR home therapy [mp] OR home care services [mh] OR home management [mp] iv. Observational study [mh] OR cross-sectional studies [mh] OR cohort studies [mh] OR longitudinal studies [mh] OR epidemiologic studies [mh] OR case-control studies [mh] OR controlled clinical trial [mh] OR randomized controlled trial [mh] OR non-randomised controlled trials as topic [mh] OR quasi-experimental study [mp] OR clinical trial [mh] OR comparative study [mh] v. i AND ii AND iii AND iv	

Publications identified through the searches were exported into reference management software (EndNote X8, Clarivate Analytics, Boston, USA). Duplicate records were excluded. The reference management file was subsequently exported to systematic review software (Covidence, Veritas Health Innovation, Melbourne, Australia) for screening. Titles and abstracts of all identified publications were screened for eligibility against the review selection criteria (i.e. eligible study design, participants, exposure and outcomes) by two reviewers (a process shared by all reviewers), independently. Remaining publications underwent full-text screening by two reviewers (a process shared by all reviewers), independently. At both stages of the process, disagreements were arbitrated by a third reviewer.

### Outcomes

The primary outcome of interest was hospital admission (i.e. admission/readmission to a psychiatric inpatient facility), which served as a proxy for SMI relapse. This outcome was selected because it is associated with a deterioration in health and social exclusion; it is also the primary focus of community psychiatric services, is widely used in mental health services research and is economically meaningful [21]. Secondary outcomes were hospital length of stay, emergency department presentations*,* crisis team referral, duration of crisis team treatment, crisis house referral, detention under mental health law and adverse events.

### Data extraction

Data from eligible publications were extracted using a customised data extraction tool. The tool collected information on study characteristics, research methodology, participant characteristics, interventions, comparators, outcomes, results and new references. Data extraction was performed by three reviewers (ML, MJ, RG), independently, with disagreements adjudicated by a fourth reviewer (AE).

### Risk of bias

The risk of bias of observational studies was assessed using the Risk Of Bias In Non-randomised Studies of Interventions (ROBINS-I) tool [22]. The ROBINS-I tool determines the risk of bias across seven distinct domains, including baseline and time-varying confounding, intervention classification, co-interventions, participant selection, outcome measurement, missing data and selective reporting bias. Two reviewers (ML, RG) independently evaluated the risk of bias and rated studies as having a low, moderate, severe, critical or unclear risk of bias. A third reviewer (MJ) was consulted if there was disagreement between reviewers.

The risk of bias of clinical trials was evaluated using the Cochrane Collaboration risk of bias tool [23]. The Cochrane tool assesses risk across seven domains, including allocation concealment, sequence generation, blinding of participants, personnel and outcome assessors, selective outcome reporting, incomplete outcome data and other sources of bias. Two reviewers (ML, RG) independently rated the risk of bias for each item as low, unclear or high risk. A third reviewer (MJ) was invited to arbitrate when consensus was not reached between reviewers.

### Data synthesis

Given the considerable methodological heterogeneity of studies and that no single comparable outcome was reported in more than three studies, results could not be combined by means of meta-analysis. The results were instead presented using narrative synthesis. This synthesis was undertaken by one reviewer (ML) and cross-checked by two others (RG, MJ).

### Registration and reporting guidelines

This review was prospectively registered with PROSPERO [CRD42017058694]. A detailed summary of the review methods is set out in the protocol, which has been published elsewhere [24]. The review is also reported in accordance with PRISMA reporting guidelines for systematic reviews [25].

## Results

### Search results

**Fig. 1:**
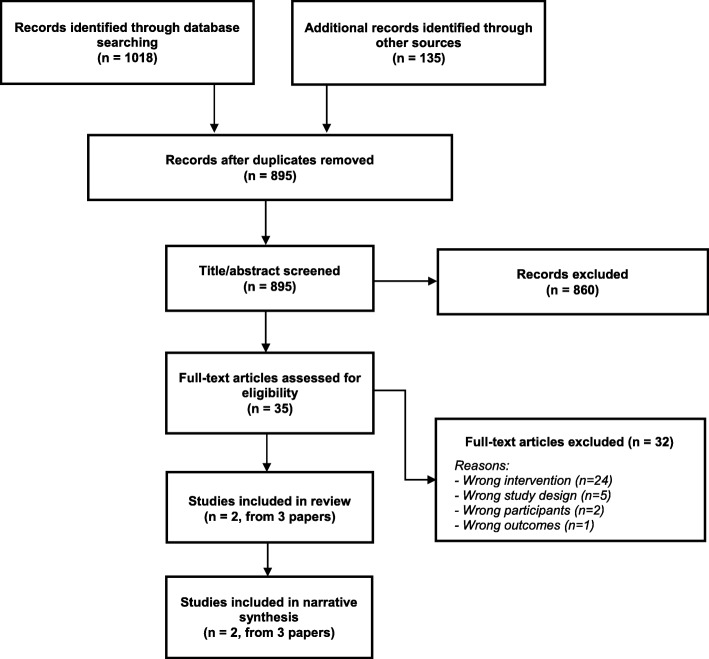
Flow chart of study selection process

The search identified 1153 publications, of which 258 duplicates were removed (Fig. 1). During title/abstract screening, 860 publications were excluded. At full-text screening, a further 32 publications were excluded, mostly because they reported the wrong intervention (*N* = 24). Three publications, reporting two discrete studies, met the selection criteria and were included in the review.

### Description of studies

Both included studies were conducted in community settings in England (Table 2). One was an observational study [26], the other a randomised controlled trial [27, 28]. Duration of follow-up was 18 months [27, 28] and 3 years [26].

**Table 2:** Characteristics of included studies

Authors	Country	Study design (duration)	Setting	Intervention	Control	Study population	Outcomes
Barr et al. [26]	England	Observational study (3 years)	Six general practices (one randomly selected practice from each health locality in an English health district)	Community mental health nurse contact with patients on the severe and enduring mental illness registers of included general practices	No contact with a community mental health nurse	*N* = 274 Inclusion criteria: participants with severe and enduring mental illness (SEMI), listed on the SEMI register of included general practices Exclusion criteria: not stated Mean age: 45.4 ± 12.2 years Gender: not specified Dropouts: not applicable	Admission to psychiatric hospital, frequency of community mental health nurse contacts
McCrone et al. [27], Muijen et al. [28]	England	Randomised controlled trial with two parallel arms (18 months)	Community setting within the Greenwich health authority	Community support team (comprising three community psychiatric nurses (CPNs), a team leader and four unqualified mental health workers). Each CPN acted as a care manager/client advocate. [None of the unqualified mental health workers provided services to the research group of 41 clients.]	Generic team (comprising six generic CPNs who worked independently, but were often attached to GP practices).	*N* = 82 Inclusion criteria: patients with a psychotic disorder (schizophrenia or affective psychosis) lasting more than 2 years, at least 2 hospital admissions in the previous 2 years, aged 18–64 years, living in the Greenwich health district Exclusion criteria: patients with primary organic disorders Mean age: 37 ± 11 years Gender: 56.1% male Dropouts: 10 (24%) patients in the intervention group and 14 (34%) patients in the control group dropped out, for the following reasons: 20 refused to participate, 2 moved away, 1 could not be found and 1 was in prison	Health service use, Global Adjustment Scale, Present State Examination, Brief Psychiatric Rating Scale, Social Adjustment Scale, patient and carer satisfaction, health service costs, accommodation costs, community psychiatric nurse costs

### Description of participants

The 2 studies involved a total of 356 patients with serious mental illness. McCrone et al. [27]/Muijen et al. [28] restricted their sample to patients with psychotic disorders, whilst Barr et al. [26] included all patients with a SMI. Participants were primarily middle-aged, and just over half were male (gender was not reported in the Barr et al. [26] study).

### Description of interventions

Both studies reported community mental health nursing as the intervention under investigation. However, there were differences between the two interventions. Barr et al. [26] examined the association between community psychiatric nurse (CPN) contact and psychiatric admission versus no CPN contact. McCrone et al. [27]/Muijen et al. [28] compared the effect of intensive CPN support, as measured by increased CPN contact time, versus traditional CPN support. Patients in the intensive support team received twice the amount of contact with CPNs than patients in the traditional CPN group. Table 2 provides additional, albeit limited details (due to insufficient reporting in the publications), of the interventions used in this study.

### Description of outcomes

The two studies reported distinct outcomes, two of which were relevant for this review: admission to hospital [26] and health service use (i.e. hospital admissions and emergency department presentations) [27, 28].

### Assessment of risk of bias

The methodological quality of Barr et al. [26] was appraised using ROBINS-I (Table 3). The study was found to be of moderate methodological quality, with six of the seven parameters having a moderate risk of bias. Of note, was the high risk of confounding, as the authors made no adjustments for confounders in their analysis. The Cochrane risk of bias tool was used to appraise the McCrone et al. [27]/Muijen et al. [28] trial, which was found to be of low methodological quality (Table 3). The study received an uncertain rating for four (allocation concealment, sequence generation, blinding of participants, blinding of assessors) of the seven parameters on the Cochrane risk of bias tool. Both studies were more than a decade old, with Barr et al. [26] published 18 years ago and McCrone et al. [27]/Muijen et al. [28] published 24 years ago. Accordingly, these studies predate the introduction of quality standards for the reporting of clinical trials.

**Table 3:** Risk of bias of included studies

Study	Risk Of Bias In Non-randomised Studies of Interventions (ROBINS-I) tool (for observational studies)						
	Confounding	Participant selection	Intervention classification	Co-intervention	Missing data	Outcome measurement	Selective reporting
Barr et al. [26]	High risk	Moderate risk	Moderate risk	Moderate risk	Moderate risk	Moderate risk	Moderate risk
Study	Cochrane risk of bias tool (for randomised controlled trials)						
	Sequence generation	Allocation concealment	Blinding (participants/personnel)	Blinding (outcome assessors)	Objective outcome	All outcomes reported	Possible biases
McCrone et al. [27], Muijen et al. [28]	Unclear risk	Unclear risk	Unclear risk	Unclear risk	Low risk	Low risk	High risk

### Effects of interventions

#### Hospital admission rates

Two studies reported psychiatric inpatient admission rates as an outcome. Barr et al. [26] reported more psychiatric admissions in patients receiving community psychiatric nurse care at 3 years follow-up compared with patients that had no CPN contact (81% vs. 19% of sample, respectively). McCrone et al. [27], on the other hand, reported fewer admissions in patients receiving intensive community psychiatric nurse support team care at 12–18 months compared with patients receiving generic CPN care (0% vs. 10% of sample, respectively). By contrast, Muijen et al. [28] reported no significant difference in the mean number of hospital admissions between the intensive and generic CPN care groups at 12–18 months (0.4 admissions in each group). It is assumed that hospital admissions in this study referred to admission to general and not psychiatric settings.

#### Emergency department (ED) presentations

One study (McCrone et al. [27] or Muijen et al. [28]) reported ED presentations as an outcome. McCrone et al. [27]/Muijen et al. [28] found patients in the generic CPN care group were more likely to present at an emergency department in the first 6 months of treatment compared with patients in the intensive CPN care group (6% vs. 3% of participants). However, there were no notable differences between the two groups at 6–12 months (3% vs. 3% of participants) and 12–18 months (7% vs. 6% of participants).

#### Other outcomes

The included studies did not report data on hospital length of stay, crisis team presentations, admissions to crisis houses, detention in hospital under mental health law or adverse events.

## Discussion

This review investigated the association between exposure to community mental health nursing and hospital admission among individuals living with SMI. The two included studies, both conducted in the UK, were rated as being of low to moderate methodological quality. The findings were also inconsistent. One study reported increased odds of admission to psychiatric inpatient facilities among patients receiving CPN care (versus no CPN care) [26]. The other study found no difference in the rates of psychiatric inpatient admissions between patients receiving generic and intensive CPN care [28]. Hence, the effect of community mental health nursing care on hospital admission (or relapse) prevention in people with SMI is inconclusive.

The paucity of studies examining the association between exposure to community mental health nursing and hospital admission in people with SMI should not be viewed as a limitation, but rather an opportunity. The review has uncovered an important evidence gap in the field, highlighting the need for methodologically rigorous research aimed at better understanding the impact of mental health nursing care [29]. We would therefore argue that the findings of our review provide an important impetus for future investment in mental health nursing research and education.

It is surprising that there have been so few relevant studies at a time when nursing workforce research in general settings has been the focus of a number of important and influential studies [30] and reviews [31]. It is also noteworthy that the studies included in our review were all from the UK. It is difficult to offer an informed insight as to why this might be. Perhaps researchers and funders do not recognise the value of better understanding the impact of the community mental health nurses on patient outcomes.

Some qualitative studies have reported that patients value the contribution that mental health nurses make to their care and treatment. In a review of 17 reports on the Australian Mental Health Nurse Incentive program, Happell and Phung [32] concluded that mental health nurses benefited the health of people with mental illness with respect to increasing access to primary care. This is in contrast to the predominantly negative findings from a qualitative study of 23 Australian mental health nurse graduates [33], in which participants reported that mental health nursing staff were uncaring, and patients were often neglected, and in some cases, mistreated.

Rather than focussing on the strengths and limitations of the existing mental health nursing workforce, a number of randomised controlled trials (RCTs) in the UK have explored whether changes in mental health nursing education (i.e. training to deliver specific evidence-based interventions) could impact patient outcomes. The evidence from these studies has been largely positive. For example, in a meta-analysis of 53 RCTs involving 2981 patients with schizophrenia, Pharoah et al. [34] found that educating mental health workers (from non-specific disciplines) to work with the families of people living with schizophrenia was effective in reducing patient relapse, increasing adherence with medication and reducing admission to hospital. Nevertheless, these studies do not provide evidence specifically related to mental health nursing. There is a clear need to better understand optimal nursing skill mix in community mental health settings. A research question of primary importance would be to understand the association between skill mix (e.g. ratio of nurses to other health professionals) in community mental health teams and patient outcomes (of which admission to acute care is used as a proxy for relapse).

### Limitations

The focus of this review was on hospital readmission of patients with SMI. The review did not include studies that focused on quality of life, recovery, reduced suicide risk, vocational needs or overall satisfaction with care—all of which are areas where community mental health nursing may have played an important role. While admission to hospital was used as a proxy measure for relapse of SMI, this may not be an accurate measure of relapse since home treatment or crisis teams have been part of standard care for people in mental health crisis in the UK since 2000. In addition, people may be admitted to psychiatric inpatient facilities for clozapine initiation, which may not represent a relapse of SMI. However, admission to hospital has been used as a measure of relapse in other studies [35] and is an objective outcome used in psychiatric services. The inclusion of crisis teams as a proxy measure for relapse might yield different results in future reviews, although the view of the authors is that generating new primary data should be the priority for future research.

## Conclusions

Mental health nurses represent over half of the global mental health workforce [36]. While nurses are often cited in policy as being pivotal members of mental health teams, this review found little evidence that exposure to community mental health nurses was associated with lower odds of being admitted to psychiatric inpatient care. Given the paucity of consistent, high-quality evidence addressing the impact of community mental health nursing on relapse in SMI and the evidence-base supporting the practice of other disciplines and branches of nursing, there is a need to build an evidence base to inform the planning of community mental health services.

## Data Availability

The datasets used during the current study are available from the corresponding author on reasonable request.

## Abbreviations

CMHN: Community mental health nurse
CPN: Community psychiatric nurse
PRISMA: Preferred Reporting Items for Systematic Reviews and Meta-Analyses
RCT: Randomised controlled trial
ROBINS-I: Risk Of Bias In Non-randomised Studies of Interventions
SMI: Serious mental illness
